# Polyphenols vs. Coronaviruses: How Far Has Research Moved Forward?

**DOI:** 10.3390/molecules25184103

**Published:** 2020-09-08

**Authors:** Simona Piccolella, Giuseppina Crescente, Shadab Faramarzi, Marialuisa Formato, Maria Tommasina Pecoraro, Severina Pacifico

**Affiliations:** Department of Environmental, Biological and Pharmaceutical Sciences and Technologies, University of Campania “Luigi Vanvitelli”, Via Vivaldi 43, 81100 Caserta, Italy; simona.piccolella@unicampania.it (S.P.); giuseppina.crescente@unicampania.it (G.C.); shadab.faramarzi@unicampania.it (S.F.); marialuisa.formato@unicampania.it (M.F.); mariatommasina.pecoraro@unicampania.it (M.T.P.)

**Keywords:** polyphenols, coronavirus, antiviral activity, papain-like protease, 3-chymotrypsin-like protease, herbal extracts

## Abstract

The epidemic, caused by SARS-CoV-2 at the beginning of 2020, led us to a serious change in our lifestyle that for about three months has confined us to our homes, far from our laboratory routine. In this period, the belief that the work of a researcher should never stop has been the driving force in writing the present paper. It aims at reviewing the recent scientific knowledge about in vitro experimental data that focused on the antiviral role of phenols and polyphenols against different species of coronaviruses (CoVs), pointing up the viral targets potentially involved. In the current literature scenario, the papain-like and the 3-chymotrypsin-like proteases seem to be the most deeply investigated and a number of isolated natural (poly)phenols has been screened for their efficacy.

## 1. Introduction

Four months after the COVID-19 pandemic declaration by the World Health Organization (WHO), the global consequences, both social and economic, are severe, despite the initial desperate health emergency is actually over. Nowadays, everyone in the world is aware of the existence of a novel coronavirus, named severe acute respiratory syndrome coronavirus 2 (SARS-CoV-2). The principal reason is the record number of countries involved, the uncountable number of cases worldwide, due to an unexpected rapidity and aggressiveness, after its first identification in Wuhan City (Hubei province, China) on the 31st of December 2019 [1].

Italy has been one of the first European countries to face and try to counteract this growing epidemic, when, at the beginning of March 2020, the Italian Government imposed the mandatory stop of all non-necessary activities and a prohibition to move out of one’s own municipality of residence, except for demonstrable health or work needs. School, university and educational activities were also stopped, permitted only remotely, in order to avoid crowds.

**Figure 1:**
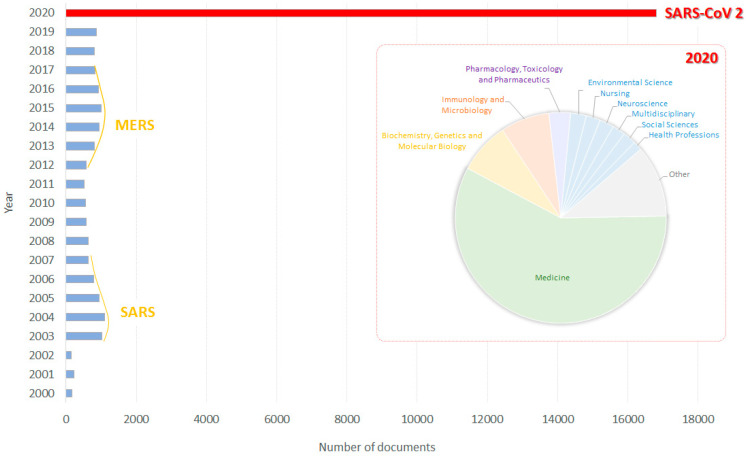
Number of documents per year dealing with the word “coronavirus.” In the red box documents by subject area published in 2020 (source Scopus Database, accessed on 1 July 2020).

What happened to scientific research in this lockdown period? To answer this question, it is enough to open the Scopus database and to digit the word “coronavirus,” limiting the search to the last two decades, keeping in mind that the other two epidemics caused by human coronaviruses (SARS) and MERS (Middle East Respiratory Syndrome) spread in 2002/2003 and in 2012, respectively [2]. The results were absolutely astonishing: if the average number of articles was quite constant until 2019, in only six months of 2020 it had a 10-fold increase, reaching about 10,500 published papers (Figure 1), and this number is expecting to increase in the next weeks.

Thus, scientific research represents the only field that had the power to overcome national restrictions, physical distancing and fear of infection, in order to contribute to global human health promotion. It is not surprising that, among all the journals considered in 2020 for publication, about 70% fall into “medicine,” “immunology and microbiology” and “biochemistry, genetics and molecular biology” subject areas, whereas chemistry papers on the topic represent only 1% (Scopus database, accessed on 1 July 2020).

As chemists with a long experience in natural products, our aim was to exploit our new and strange “smart working” condition, far from our laboratory benches and our experiments, to build up a review article, dealing with experimental evidence of the role of polyphenol compounds, both in pure forms and in mixture in plant extracts, as antiviral agents with a potential role against different species of coronaviruses. To better deepen the discussion, a brief introduction on viral replication and on the different targets involved will be provided.

## 2. Viral Structural Features and Replication

**Figure 2:**
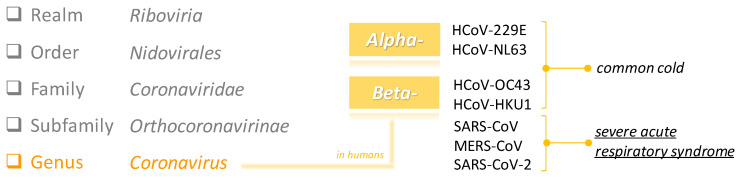
Taxonomy of coronaviruses (CoVs) affecting humans.

Coronaviruses (CoVs) are enveloped positive-sense single-stranded RNA viruses, which owe their name to the characteristic crown-like aspect (100–150 nm virion diameter) they show when observed by an electron microscope. From a taxonomy point of view, they belong to the *Coronaviridae* family, *Orthocoronavirinae* subfamily, which includes four coronavirus genera: *Alpha*-, *Beta*-, *Delta*- and *Gamma*- [3]. CoVs are able to infect both humans and animals, including birds and mammals, targeting epithelial cells of the respiratory and gastrointestinal tract. To date, seven CoVs have chosen humans as their hosts, causing mainly respiratory diseases with a different degree of severity (Figure 2) [2,4].

**Figure 3:**
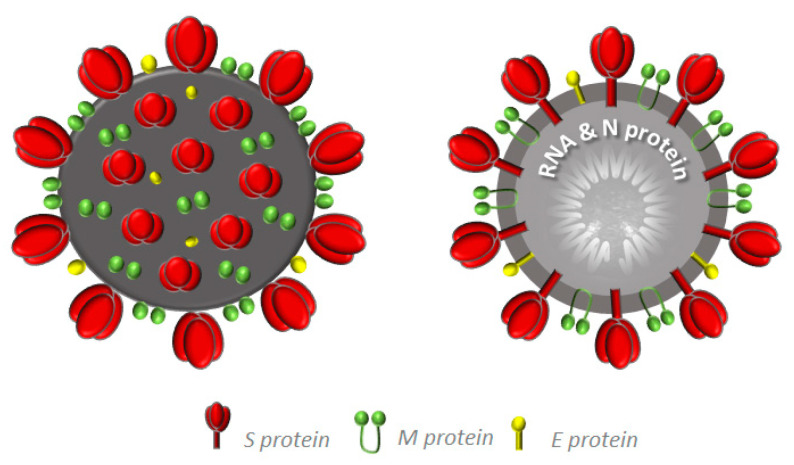
CoVs’ structural proteins.

The genome of SARS-CoV-2 was found to be similar to those sequenced for SARS and MERS. Thus, all the information previously acquired has been very useful to describe most of the proteins and their role in virus features and virus-host interaction [5,6]. The genomic RNA size is about 30 kb, organized in Open Reading Frames (ORFs), which encode four main structural proteins: Spike (S), Membrane (M), Envelope (E) and Nucleocapsid (N) (Figure 3) [7].

The S protein is a trimeric transmembrane glycoprotein, formed by the amino-terminal portion (S1, N-exo), which mediates the binding to the receptor, and the carboxy-terminal portion (S2, C-endo), responsible for the fusion between the virus and the host cell before its entrance [8].

The M glycoprotein is the most abundant, placed among S proteins, characterized by three transmembrane domains with the N- and the C-terminal domains outside and inside the membrane, respectively [9]. It defines the virus shape and represents the central driver of the virus assembly [10]. It has been demonstrated that M can assume two conformations, named M_LONG_ and M_COMPACT_, associated with a different degree of rigidity that can convert each other, thus influencing the membrane curvature [11].

The E protein is smaller than the others, but can oligomerize and create ion channels, acting as a viroporin. It has been shown to play key roles in the virus life cycle, from assembly to release [9,12,13].

The N protein is associated with viral RNA and is expressed in hosts at the beginning of infection. It is considered a multifunctional protein, in that it is able to cooperate with the other above-mentioned structural proteins and also with host’s proteins, improving the efficiency of virus transcription and assembly and being crucial in viral pathogenesis [14].

**Figure 4:**
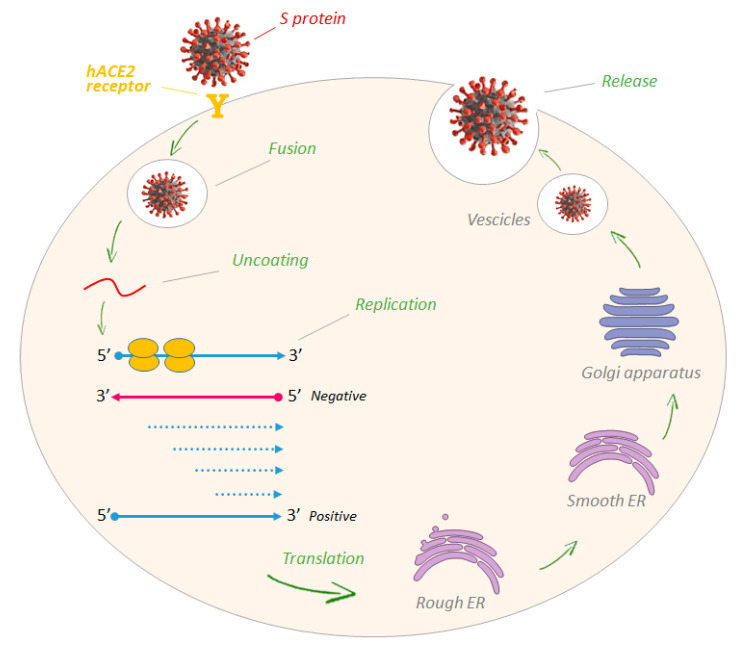
Viral replication scheme.

The viral replication cycle is schematized in Figure 4. The first approach of the virion to the host cell is mediated by the S protein. In particular, the receptor binding domain (RBD), a stretch of about 200 amino acids located at the S1 end, has the function to bind specific receptors, which vary from one group to another and often among members of the same group, on the surface of the host cell [15]. SARS-CoV and SARS-CoV-2 specifically bind the hACE2 protein (Human Angiotensin Converting Enzyme 2), whereas MERS-CoV utilizes DPP4 (Dipeptidyl Peptidase 4). Depending on the viral strain, virus entry can take place in two ways: via endocytosis, involving cathepsins, or via fusion of the viral envelope with cell membrane, mediated by glycoprotein S using extracellular proteases, such as TMPRSS2 (Transmembrane Protease Serine 2) [16,17].

Once inside the cell, the virus gets rid of the capsid and releases the genetic material, thanks to lysosomal enzymes. Then, the replication of the viral genome and the synthesis of functional and structural viral proteins occur. Among these mature proteins are an RNA-dependent RNA polymerase (RdRp), an RNA helicase and other enzymes. The replicase translates the viral positive RNA in negative RNA, which is used to produce a series of subgenomic mRNAs and the viral genome [18]. Two cysteine proteases, papain-like protease (PLpro) and 3-Chymotrypsin-like protease (3CLpro), encoded by ORF1a, play a key role in some CoVs’ replication (release and liberation of other nonstructural proteins). Although their main function is to process the viral polyprotein, PLpro also strips ubiquitin and interferon-stimulated ubiquitin-like protein (ISG15) from host-cell proteins, to let coronaviruses evade the host innate immune responses. Like 3-chymotrypsin-like protease (3CLpro), it is synthesized as a precursor protein, and then cleaved to give rise to mature active proteins and its structure is well-maintained across the CoV genera. Thus, together with 3CLpro, it could be an attractive target for antiviral drugs [19,20,21]. The genomic RNA and viral proteins are assembled into virions on membranes that are located between the endoplasmic reticulum (ER) and the Golgi apparatus (ER-Golgi intermediate compartment-ERGIC); finally, they are transported via vesicles and released out of the cell [18].

## 3. (Poly)Phenol Compounds vs. CoVs: Targets Involved

Taking into account the aforementioned CoVs’ structural features and replication, it is likely to hypothesize that the most promising targets to counteract their infection, which nowadays can be counted among the leading causes of morbidity and mortality both in developed and developing countries, include 3CLpro and PLpro proteases, besides hACE2 receptor and other nonstructural proteins, such as RdRp polymerase, helicase and N-7 methyltransferase (N7-MTase).

Natural compounds have always drawn the attention of researchers, in that they are able to exert a very broad range of appealing biological activities, whose pharmacological and/or toxicological effects can be exploited in preventing or counteracting a number of health-related problems, and also as leading compounds for the synthesis of derivatives with enhanced bioactivity [22,23]. Phytochemicals have a long history in the treatment of infectious diseases, including those of viral origins, which dates back to the end of the Second World War to keep going with unceasing in vitro and in vivo updates regarding the antiviral capacity of several medicinal plant extracts and pure bioactive compounds against a wide range of microorganisms [24].

**Figure 5:**
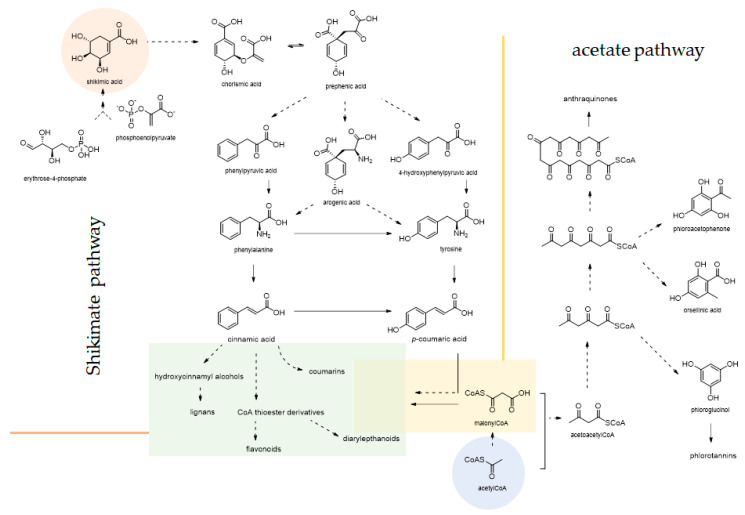
Phenols and polyphenols, which are shown to exert antiviral activity, originate from shikimate and acetate pathways. Most of them are the result of an intertwining of the two pathways. The shikimate pathway begins through the condensation of phosphoenolpiruvic acid (PEP) and erythrose-4-phosphate (E4P) by 3-deoxy-D-arabino-heptulosonate 7-phosphate synthase to produce 3-deoxy-D-arabino-heptulosonic 7-phosphate acid, which is promptly converted by the 3-dehydroquinate synthase in 3-dehydroquinic acid. This latter undergoes dehydration by the 3-dehydroquinate dehydratase to provide 3-dehydroshikimic acid, which is converted in shikimic acid through the action of shikimate dehydrogenase (SDH). Many intermediates of the shikimate pathway can be branch points leading to different C_6_C_0_, C_6_C_1_, C_6_C_2_ and C_6_C_3_ aromatic compounds, which can further act as precursors of more complex metabolites. This is the case of gallic acid, from which condensed and hydrolysable tannins are formed. The conversion of shikimic acid in chorismic acid by another PEP moiety condensation favored aromatic amino acids biosynthesis. Indeed, chorismic acid is converted by chorismite mutase via a pericyclic Claisen rearrangement, in prephenic acid, whose conversion to L-phenylalanine and L-tyrosine may occur via two alternative pathways. L-tryptophan is biosynthetized when chorismic acid is converted to anthranilic acid by anthranilate synthase. The nonoxidative deamination of L-phenylalanine by phenylalanine ammonia-lyase (PAL) forms *trans*-cinnamic acid, which is the parental compound for the biosynthesis of phenylpropanoids (e.g., hydroxycinnamic acids, lignans and coumarins), aromatic polyketide (e.g., diarylheptanoids, flavonoids, stilbenes, flavolignans and isoflavonoids) and terpenoid quinones. The acetate pathway could form fatty acids by fatty acid synthase (FAS) catalysis or polyketide systems by different polyketide synthases. Poly-β-keto chain is highly reactive and could undergo intramolecular aldol or Claisen reactions supplying. Phloroglucinol derives from a polyketomethylene chain formed by three malonyl-CoA units, whereas anthraquinones are from a 16-carbon poly-β-keto chain.

Among phytochemicals, phenols and polyphenols have been broadly investigated for their role in different stages of viruses’ attack, from their entry into hosts to their replication and transcription processes, until their releasing. Phenols and polyphenols are mainly from acetate (via malonate) and/or shikimate pathways (Figure 5). These latter occur in plants to produce bioactive metabolites, useful to the producer plant thanks to their deterrence/anti-feedant activity, toxicity or the involvement in protection and defense systems. A very recent review article includes this class of compounds among potential agents against CoVs [25].

### 3.1. Papain-Like Protease (PLpro)

**Figure 6:**
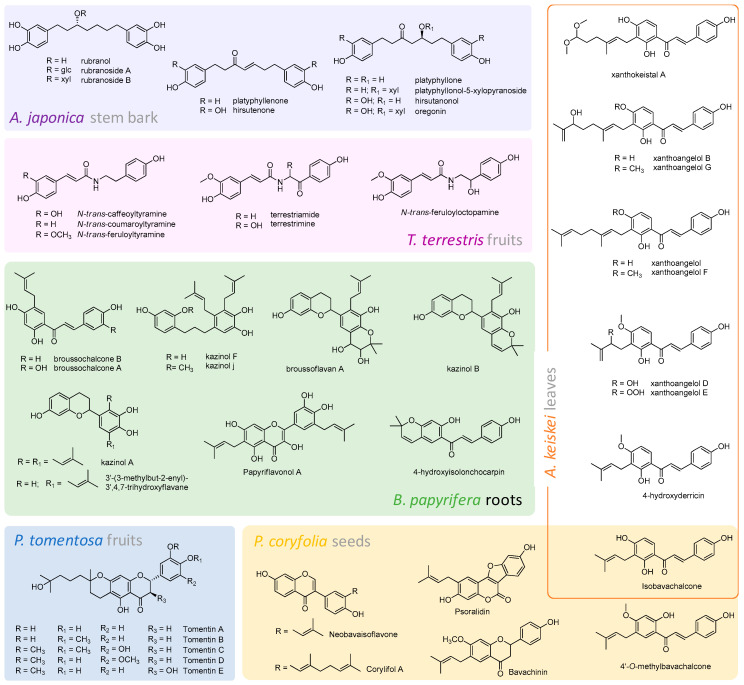
Chemical structures and related plant sources of some promising natural compounds with PLpro inhibiting activity.

In recent years, a number of studies about the inhibition of PLpro by pure compounds isolated from plant sources has been carried out. In this context, *Psoralea corylifolia* L. has been considered. This plant, belonging to the Leguminosae (Fabaceae) family, is an annual herb of the plains of India [26], and its seeds are used as food additive in many countries, especially South Korea. *P. corylifolia* has a great biological importance, thanks to its chemical composition. Indeed, its constituents have been found to exhibit a plethora of activities, including antioxidant, antibacterial, anti-inflammatory and antidepressant ones [27]. Phenolic glycosidase inhibitors have been also described from this species; in particular, among them, psoralidin is the principal contributor to the α-glucosidase inhibition. It is worth of note that many animal viruses have an external envelope consisting of viral glycoproteins; these are often necessary for the viral life cycle and use cellular machinery for synthesis. The result is alteration of cell–cell or cell–virus recognition processes [28]. An ethanol extract prepared from the seeds of *Psoralea corylifolia* L. showed a half maximal inhibitory concentration (IC_50_) value of 15 μg/mL in inhibiting SARS-CoV PLpro [27]. This promising capacity led the authors to the isolation of six flavonoid compounds, through a bioactivity-guided fractionation of the crude extract, whose identity was established by spectroscopic and spectrometric techniques (Figure 6). All pure compounds inhibited PLpro in a dose dependent manner. In detail, psoralidin (a coumestan derivative) was the most effective inhibitor with an IC_50_ of 4.2 µM. Chalcones (isobavachalcone and 4′-*O*-methylbavachalcone) exhibited a significant inhibition degree (IC_50_ = 7.3 and 10.1 µM), higher than isoflavones (neobavaisoflavone and corylifol A). The flavanone bavachinin was the least active, with an IC_50_ of 38.4 µM. Furthermore, the investigation of the inhibition kinetics by Lineweaver–Burk plots revealed that isobavachalcone and psoralidin were mixed type I inhibitors, in that they were able to differently bind to the free enzyme and to the enzyme-substrate complex, and in particular the link to the free enzyme was slightly stronger [27].

α-Glucosidase inhibition capability was also demonstrated for some polyphenols from the roots of the *Broussonetia papyrifera* L. tree. It belongs to the Moraceae family, also known as paper mulberry, and is widely spread in Asia and Pacific countries such as China, Thailand and the USA. Several plants of genus *Broussonetia* have gained attention, because of health beneficial activities ascribed to bioactive compounds present in fruits, bark, leaves, flowers and roots, which defined their uses in Chinese traditional medicine as antioxidant, anti-inflammatory, antiplatelet, anti-tyrosinase, antimicrobial and antinociceptive remedies [29]. In particular, as regards the inhibitory activity against α-glucosidase, a chloroform extract from the roots of *B. papyrifera* exhibited a significant effect with IC_50_ of around 9.3 μg/mL. The subsequent phytochemical study of this extract led to the isolation of 12 polyphenols, whose structural features allowed the authors to conclude that the promising activity could be strictly related to the presence of prenyl groups in the molecule [30]. Indeed, prenylation, which is due to incorporation of isoprenic moieties from mevalonate pathways, improved the lipophilicity and bioactivity of polyphenols. Thus, these latter could enhance their affinity to biological membranes and the interaction with target proteins [31].

Some years later, these results have encouraged the investigation of *B. papyrifera* polyphenols antiviral activities against CoVs cysteine proteases [32]. The compounds isolated were characterized by means of spectroscopic and spectrometric techniques such as broussochalcone A and B, 4-hydroxyisolonchocarpin, papyriflavonol A, 3′-(3-methylbut-2-enyl)-3′,4′,7-trihydroxyflavane, kazinol A, B, F and J and broussoflavan A (Figure 6). All tested polyphenols showed a poor inhibitory activity against MERS-CoV PLpro, whereas they proved to have a dose-dependent inhibitory effect on SARS-CoV PLpro in comparison with the other cysteine proteases. Among them, the prenylated derivative papyriflavonol A exhibited the highest inhibitory effects on SARS-CoV PLpro (IC_50_ = 3.7µM). To investigate the contribution of the two prenyl groups, the researchers compared its efficacy with that of quercetin, quercetin-*β*-galactoside and kaempferol, which shared the same flavonol nucleus with papyriflavonol A, observing a 2.3-, 14- and 4.4-fold decrease in the inhibitory potency, respectively. Thus, they hypothesized the formation of strong hydrophobic interactions between the enzyme and the prenyl chain, pivotal in exerting the PLpro inhibition. Furthermore, considering the number of hydroxyl groups on the molecular skeleton, the authors concluded that the increase of the hydroxylation degree was positively correlated with the inhibitory effect, whereas the presence of a saccharide residue resulted in a lower activity. On the contrary, the presence of the prenyl group as a closed ring caused a significant decrease in the protease inhibitory activity, and this was observed especially among chalcone derivatives [32].

Chalcone compounds were also the main actors responsible for the antiviral efficacy of *Angelica keiskei* (Miq.) Koidz. This plant, belonging to the Umbelliferae (Apiaceae) family, is also called “Myeong-Il Yeob” in Korea or “Ashitaba” in Japan, which means “the leaves of tomorrow.” It is also called “Shin-Sun Cho,” which refers to “a precious herb used by God.” Its multiple health-related properties made its aerial parts useful to treat several diseases in folk medicine in Japan, where it is marketed as a food and additive in drinks [33]. Indeed, several bioactive compounds, such as coumarins (e.g., psoralen, bergapten, xanthotoxin and isopimpinellin), alkyl chalcones (e.g., isobavachalcone, 4-hydroxyderricin, xanthoangelol, -F, -D, -E, -B, -G and -A) and flavonoids were identified [34,35,36], and have showed antioxidant, antidiabetic, antihypertensive and cancer chemopreventive effects. Recently, Park et al. [37] isolated nine alkylated chalcones (Figure 6) from *A. keiskei* leaves, extracted with ethanol and further fractionated by means of liquid-liquid partitioning and chromatographic techniques. Pure compounds were tested towards SARS-CoV PLpro and all showed a dose-dependent effect, acting as reversible inhibitors. Xanthoangelol E exhibited the most potent inhibitory activity against SARS-CoV PLpro, with an IC_50_ value equal to 1.2 µM that was even 40-fold lower than the other analogues. These results allowed the authors to hypothesize that the –OOH group on the substituted hemiterpene could be crucial in the binding to the enzyme. From the same extract, four coumarins were also isolated, but they showed IC_50_ values greater than 200 µM [37].

Chinese traditional medicine offers another polyphenol rich plant, *Paulownia tomentosa* Steud. (Scrophulariaceae family), whose fruits’ consumption has been associated to a decrease in the frequency of asthmatic attacks, hypotensive effects and the ability to regenerate hair and stimulate the scalp. *C*-geranylated flavonoids were claimed as its main bioactive constituents, and until now, 76 compounds with this structural feature were isolated and studied for their biological potential as antioxidants, anti-inflammatory and cytotoxic agents and enzyme inhibitors. In some cases, also structure–activity relationships and application perspectives have been established [38]. As regards PLpro inhibition, Cho et al. [39] purified 12 geranylated flavonoids from the fruits of *P. tomentosa*, through methanol maceration at room temperature and following chromatographic methods. Nuclear Magnetic Resonance (NMR) spectroscopy revealed an unusual 3,4-dihydro-2*H*-pyran moiety as a common skeleton for five of them (tomentin A-E; Figure 6), whereas the other ones shared a classic *C*-geranyl chain at C-6 position of the flavanone nucleus (3′-*O*-methyldiplacol, 4′-*O*-methyldiplacol, 3′-*O*-methyldiplacone, 4′-*O*-methyldiplacone, mimulone, diplacone and 6-geranyl-4′,5,7-trihydroxy-3′,5′-dimethoxyflavanone). A fluorogenic in vitro assay was used to estimate PLpro activity in the presence of these molecules, which showed dose-dependent inhibitory effects. In particular, tomentins proved to be the most promising compounds. Thus, the cyclic structural arrangement could be responsible for the more pronounced enzyme inhibition, with respect to that exerted by parent compounds.

Also derived from and widely used in Chinese medicine [40], *Tribulus terrestris* L. (Zygophyllaceae family) was investigated for its richness in cinnamic amides, with the awareness that several synthesized amide derivatives were able to inhibit PLpro [41,42]. A methanol extract prepared from *T. terrestris* fruits was the starting point to isolate six cinnamic amides: *N*-*trans*-caffeoyltyramine, *N*-*trans*-coumaroyltyramine, *N*-*trans*-feruloyltyramine, terrestriamide, *N*-*trans*-feruloyloctopamine and terrestrimine (Figure 6). A dose-dependent behavior was observed in the enzyme inhibition. Lineweaver–Burk and Dixon plots were successfully used to define their mechanism of action, described as mixed type, with terrestrimine as the most effective compound (IC_50_ = 15.8 μM). The activity was putatively ascribed neither to the ferulic acid moiety, in that, considered as pure reference molecule it proved to be inactive, nor to the cathecol A ring. In fact, both *N*-*trans*-caffeoyltyramine and *N*-*trans*-coumaroyltyramine showed similar activities. Instead, for a higher inhibitory effect, the presence of a keto and/or hydroxyl group at C7′/C8′ positions appeared to be essential [43].

In the search of other phenolic compounds to be used as effective agents counteracting PLpro activity, Park et al. [44] were the first authors to take into consideration diarylheptanoids from *Alnus japonica* Steud., a tree belonging to the family of Betulaceae. These molecules, characterized by a 1,7-diphenylheptane skeleton, had already exhibited an excellent antiviral effect, especially platyphyllone, against KBNP-0028 (H9N2) avian influenza virus [45]. Park et al. expressed and purified PLpro enzyme in *Escherichia coli* and tested nine diarylheptanoids (Figure 6), isolated from an ethanol extract of the plant stem bark and characterized by one-dimensional (1D) and two-dimensional (2D)-NMR experiments, together with Electrospray Ionization Mass Spectrometry (ESI-MS), both in positive and in negative ion modes. The diarylheptanoids proved to be reversible inhibitors, in that their concentration increase was associated with a rapid reduction of PLpro activity. Among them, hirsutenone was of note, as it showed the lowest IC_50_ value (4.1 μM), even compared with curcumin (5.7 μM), which was used as positive standard. The structural features that could be positively related to its bioactivity are the catechol moiety and the *α, β*-unsaturated carbonyl group. In particular, the latter was thought to covalently link the cysteine residue in the enzyme active site, acting as a Michael acceptor and undergoing nucleophilic addition [44].

### 3.2. 3-Chymotrypsin-Like Protease (3CLpro)

As mentioned above, 3CL-pro, also known as main protease (Mpro), was found to be highly conserved among CoVs, including SARS-CoV-2, which revealed a 99% sequence identity with the previous SARS-CoV [46]. For this reason, in the search for new anti-CoV agents, it is the most studied target, together with PLpro. Thus, it is not surprising that a number of natural compounds were tested for their inhibitory properties towards both targets. Purified molecules from *B. papyrifera* L., previously described as regards PLpro (Figure 6), investigated by Park et al. [32] are a good example. However, the effect towards SARS-CoV 3CL-pro was found to be less pronounced, in that IC_50_ values were in the range 30.2–233.3 μM, and none of them proved to be selective for MERS-CoV enzyme. 

The joint approach vs. PL and 3CL proteases involved also *A. keiskei*. In particular, the EtOAc fraction, deriving from the leaf ethanol extract by liquid-liquid extraction, showed a 75% inhibition of SARS-CoV 3CLpro at a 30 µg/mL dose level. The compounds purified therefrom were able to decrease the enzyme activity in a dose-dependent manner. In particular, chalcones once again were the most efficient, with xanthoangelol E (Figure 6) being the main inhibitor on cell-free *trans*-cleavage activity. The same compounds were also tested by cell-based cis-cleavage assay and their effects were enhanced about twice. As for the PLpro results, structure-activity relationship deductions were hypothesized [37].

During the 2002–2003 SARS epidemic, because of the absence of drugs able to counteract the respiratory syndrome and its related negative effects, the Health Ministry of China recommended some preventive formulas containing herbs used in Chinese traditional medicine. The species *Houttuynia cordata* Thunb, a perennial herbaceous plant of the Saururaceae family, native to Southeast Asia, was embraced among the ingredients of these formulas. Despite that it grows spontaneously, especially in southern China and Japan, nowadays it is cultivated all over the world as an ornamental plant, thanks to its versatility. Numerous beneficial properties have been ascribed to this plant, e.g., antiviral, antitumor, antimicrobial, anti-inflammatory and antioxidative. The rationale behind this recommendation was the occurrence of a number of studies in the literature that highlighted its antiviral activity. Indeed, as later reviewed by Fu et al. [47], this plant has an excellent potential for the development of antiviral agents against SARS, dengue, herpes simplex virus-1 (HSV-1), influenza virus and human immunodeficiency virus infections (HIV). A significant inhibition of 3CLpro, mediated by a water extract of this plant, was reported, together with its safety, proved by the absence of oral acute toxicity in animals [48].

*Rheum palmatum* L. is an another example of Chinese traditional herb, whose different extracts, or fractions derived therefrom, exhibited a moderate activity against SARS-CoV, inhibiting cysteine enzyme 3CLpro [49], as well as against other viruses, such as cosakievirus B_3_ [50], Japanese encephalitis virus [51] and hepatitis virus B [52]. Other different Chinese herbal extracts were also tested in the last decade to evaluate an antiviral activity focused on 3CLpro. Indeed, methanol extracts of *Cibotium barometz* L. rhizome and *Dioscorea batatas* tuber showed a comparable efficacy with IC_50_ value of 39 and 44 µg/mL, respectively [53]. Unfortunately, the lack of a systematic phytochemical study of these extracts prevents the possibility of addressing the observed activity to a particular class or sub-class of compounds.

**Figure 7:**
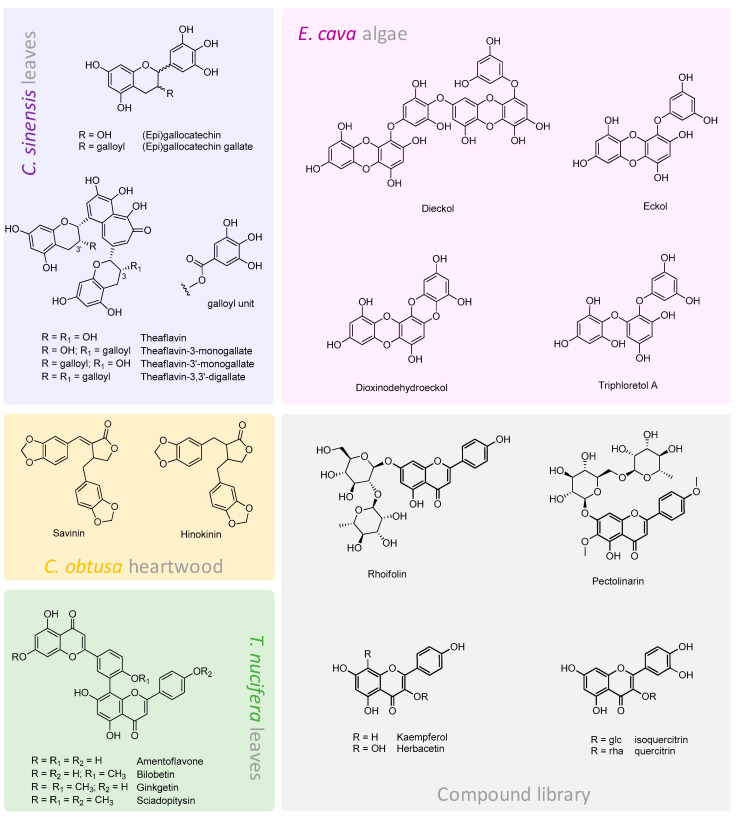
Chemical structures and related sources of some promising natural compounds against 3CLpro.

Contrariwise, an attempt to link the inhibitory capacity to the presence and abundance of certain metabolites in crude extracts has been the driving force in the investigation about different kinds of tea beverages. Thus, due to the leading role in Chinese history of tea made with *Camellia sinensis* L. leaves, and also due to its great popularity and consumption worldwide [54], it was particularly interesting to read about the different 3CLpro inhibitory potential of oolong, green, black and Puer teas. The four water infusions had a different composition in bioactive compounds. In fact, oolong tea’s chemical constituents were similar to green tea; instead, theaflavins, formed by enzyme-catalyzed oxidative dimerization of catechins during manufacturing processing [55], are particular abundant in the black tea and consist of theaflavin, theaflavin-3-monogallate, theaflavin-3’-monogallate and theaflavin-3,3’-digallate (Figure 7). The results of the antiviral activity tests showed that black and Puer tea exhibited a greater efficacy towards 3CLpro inhibition with IC_50_ values equal to 75 and 25 µg/mL, respectively [56]. In line with these results, in the same paper the authors demonstrated that pure theaflavins were much more effective than catechin monomers. Moreover, they underlined the role of the gallate group in the interaction with the enzyme active site, which resulted in an increased activity. The importance of gallate substituent have also been strengthened by Nguyen et al. [57], through a combined approach involving in vitro assays and molecular docking simulations. In fact, in their investigation gallocatechin and epigallocatechin gallate, bearing a gallic acid moiety linked at 3-OH position, inhibited 3CL-pro more effectively than epigallocatechin (85% and 91% vs. 5.4%) and other flavonoids.

In order to describe how different functions on the basic scaffold of flavonoids can increase, or not, the activity against SARS- and MERS-CoV 3CLpro, a library of compounds has been evaluated, both experimentally and computationally. The authors found that herbacetin, rhoifolin and pectolinarin (Figure 7) were the most promising SARS protease inhibitors and hypothesized that the presence of the additional hydroxyl group at C-8 position in herbacetin, compared to kaempferol, could be the key structural feature to guarantee a major binding force with the enzyme. Furthermore, as regards the presence of a saccharide moiety, they explained the greater activity against MERS-CoV 3CLpro of isoquercitrin (quercetin 3-*O*-β-d-glucoside) with respect to quercitrin based on the formation of a strong hydrogen bond mediated by the hydroxymethyl group of the glucose [58,59].

If a number of ubiquitous flavones (e.g., luteolin and apigenin) showed a moderate or poor efficacy in 3CLpro inhibition, the activity was highly enhanced in the case of biflavones. In this context, Ryu et al. [60] took into consideration an ethanol extract from the leaves of *Torreya nucifera* L., a Taxaceae tree used in traditional Asian medicine. Among the investigated compounds, isolated and identified by means of spectroscopic techniques, amentoflavone stood out for its strong inhibitory potential (IC_50_ = 8.3 µM), which was about nine-, four- and five-fold greater than the other biflavones, which were characterized by a higher methoxylation degree (Figure 7). The discussion was enriched by the comparison with its commercial flavone monomer (apigenin), which exhibited a 34-fold decreased activity, further supported by computational docking analyses.

The marked improved activity of dimer compounds was also highlighted in phlorotannins (Figure 7), isolated from the edible brown algae *Ecklonia cava*, endemic in the subtidal regions of Jeju Island, Korea. Indeed, Park et al. [61] reported for the first time that these compounds were able to inhibit 3CLpro with a competitive mechanism. Above all, dieckol, formed by two eckol moieties linked by a diphenyl ether, proved to be about three-fold more active than eckol, with IC_50_ values of 2.7 and 68.1 µM in *trans*- and *cis*-cleavage assays, respectively.

To the best of our knowledge, the only paper dealing with lignans’ specific efficacy against 3CLpro focused above all on dibenzyl butyrolactones savinin and hinokinin (Figure 7), isolated from the ethyl acetate extract of the heartwood of *Chamaecyparis obtusa* var. *formosana*. Despite the very close similarity between the two structures, savinin proved to be more effective. It has been hypothesized that, due to the C7-C8 double bond in this latter, the conjugation degree assures a planar structure, capable to better bind the enzyme [62].

### 3.3. Virus Entry: S Protein/hACE2 Receptor Interaction

As above mentioned, the S protein is the main actor in the initial binding of the virus to the host cell, before the entry via fusion of the viral envelope with the cell membrane. On the other side, ACE2 represents the functional receptor [63]. Thus, preventing their interaction should avoid the viral infection upstream.

Among 312 Chinese medicinal herbs, sub-divided in 32 families, some authors showed that aqueous extracts of those belonging to *Nelumbonaceae*, *Labiatae*, *Magnoliaceae*, *Oleaceae*, *Lauraceae* and *Polygonaceae* families were able to considerably block the S protein-ACE2 binding, with the last being the most active [64]. Anthraquinones, e.g., emodin, are known to be representative compounds of this family [65] and could be likely considered the bioactive compounds responsible for the inhibition of S/ACE2 interaction. Emodin, whose activity was dose-dependent, proved to be more active than bare anthraquinone and 1,4-bis-(1-anthraquinonylamino)-anthraquinone, suggesting that the main actors of the inhibition were the functional groups and not the molecular skeleton itself [64].

### 3.4. Other Less Investigated Targets: Focus on the Viral Replication

In the search of effective natural compounds able to elicit their antiviral activity suppressing CoV’s replication, nonstructural proteins (nsp) seemed to be promising targets, although until now they have been poorly studied in respect to the other targets, previously discussed. In particular, the RNA-dependent RNA polymerase (nsp 12) is responsible for the genome replication and transcription [66], the NTPase/helicase nsp 13 unwinds duplex RNA into two single-stranded nucleic acids [67], and is able to translocate along the nucleic acids by hydrolyzing ATP [68], whereas nsp 14 acts as an N7-MTase and is involved in RNA cap formation [69].

**Figure 8:**
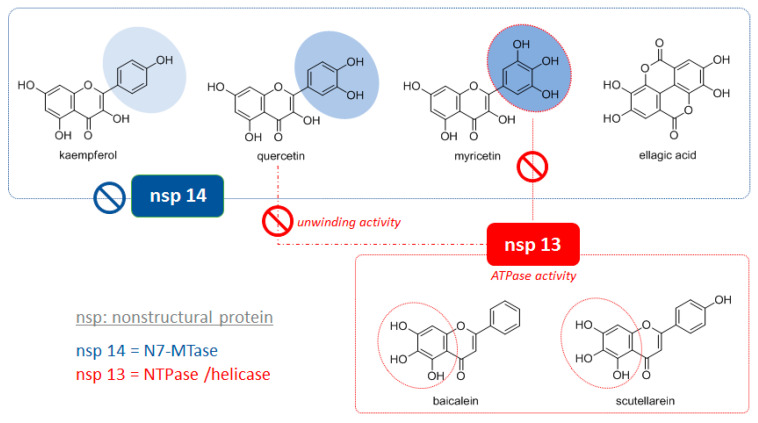
Chemical structures of polyphenols active on CoV nonstructural proteins involved in replication. In the blue box compounds able to inhibit nsp 14 (N7-MTase) are reported. In the three flavonols the chemical feature likely responsible for the activity (ring B) is highlighted. In the red box compounds acting vs. nsp 13 are reported. They share with myricetin the same mode of action, which is different from quercetin (see text for details).

Apart from ellagic acid, flavonols showed an interesting inhibitory activity towards nsp 14, as follows: myricetin > quercetin > kaempferol [70]. They differentiate for the oxidation degree of ring B. Therefore, it could be reasonable to assume that it is the key structural feature and that the higher the number of hydroxyl groups the higher the effectiveness (in Figure 8, this finding is depicted by the circles with different shades of blue). Myricetin appeared to have an important role also related to nsp 13, through inhibition of the ATPase activity, the same mechanism hypothesized for the flavone scutellarein [71], with which it shares a pyrogallol-like function (red circles in Figure 8). Baicalein, very similar to the latter in its chemical structure (Figure 8), also proved to inhibit the ATPase activity of nsP13 by more than 60% with an IC_50_ value of 0.47 ± 0.09 µM [72]. Instead, the mode of action of quercetin is different, as it selectively inhibited the duplex DNA unwinding activity in micromolar range [73].

## 4. Conclusions

**Figure 9:**
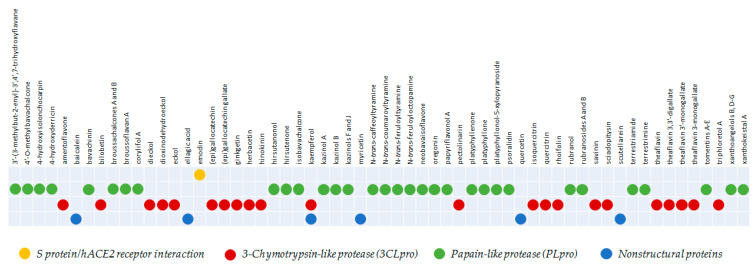
A summarizing picture of the different compounds able to interfere with the viral infection, acting vs. four main targets, highlighted in different colors.

How far has research on polyphenols vs. coronaviruses moved forward? Despite the huge number of scientific papers dealing with the coronavirus research field, information about the role of phenols and polyphenols as effective molecules, able to counteract viral infection, is still limited. Figure 9 aims to give an immediate and summarizing picture of all the different compounds tested in in vitro assays vs. four viral targets. In particular, it clearly shows that the main focus is represented by the inhibition of PLpro and 3CLpro, against which they seemed to be promising, both in pure form and in mixture. Considering isolated compounds’ identity, it appears clear that common flavones and flavonols are the main actors against 3CLpro, together with flavan-3-ols and their condensed derivatives. The high degree of cyclization and unsaturation, which also increases the compounds’ planarity, provides the good efficacy of tetracyclic lignans and phlorotannins. Phenylpropanoid derived compounds, preserving acyclic structural features of bridges among aromatic rings, promptly act vs. PLpro. As assertively discussed throughout the text, further structural elements (e.g., prenyl chains) appeared to enhance antiviral efficacy; however, data available today must be taken with a grain of salt, first of all due to the lack of a deepen experimental investigation that focuses the bioactivity evaluation towards both the proteases. It is certain that the major interest towards these proteases as attractive targets could be likely explained considering that their structures seem to be well-maintained across the CoV genera. Screened molecules could be favorably screened for and used in the future as new effective antiviral agents, alone or as supplements in combination with conventional antiviral drugs. Furthermore, the rationalization of key structural features in exerting the bioactivity could be exploited in the rational design of novel synthetic drugs with enhanced effectiveness. Needless to say, the evaluation of clinical relevance will be mandatory and also a critical evaluation of chances for translation into therapeutic practice, which currently still seems lacking.

During the last two decades several plants of Chinese traditional medicine have gained much attention. However, the main drawback of some of these studies is the lack of a chemical investigation of the extracts’ constituents and of their standardization and/or titration. This finding prevents the rationalization of their mode of action and the evaluation of their safety profile.

In light of the above and as future hopeful perspective, this review article will be a useful starting point to encourage researchers to pursue this fascinating and challenging topic, paving the way for a deeper investigation on the role of phenols and polyphenols in counteracting CoV’s infection, also supported by pharmacokinetic and toxicological data.
